# The International Medical Congress

**Published:** 1886-03

**Authors:** B. H. Catching


### THE INTERNATIONAL MEDICAL CONGRESS.

In answer to a query from a physician in Paris, as to whether the International Medical Congress will be held in this country in 1887, the *Medical Record*, January 9th, says "If we were to give to the foregoing inquiry an answer unreservedly in the affirmative, we should be misrepresenting matters to our Parisian confreres. It is announced, positively and officially, that there is to be an International Medical Congress, but we must add to this that the American profession is seriously divided over the matter of its managenent, and that a considerable part of the best men of the profession have been excluded or have been forced, in self-respect, to withdraw from all connection with it.

"We are glad to state, however, that efforts for a compromise are now being made, and that there are a number of prominent gentlemen in this city, who have heretofore held aloof from the Congress, who are willing to join hands with the present management if such concessions are made as will enable them to do this properly.

"We shall be able, probably in a fortnight, to say whether these hoped-for arrangements have been made, and we shall make the announcement to our French confreres at that time.

"If they are not made, and the present dissensions and disarrangements continue, we cannot conscientiously advise foreign physicians to come over here."

While we regret to be compelled to do so, we can only characterize the above as a wilful and deliberate effort on the part of our contemporary to injure the Congress, because a few distinguished leaders in some of our eastern cities so desire.

We most emphatically say, that "*a considerable part of the profession have*" NOT "*been excluded;*" those who are out in the cold are there because they choose to be—they resigned the positions to which they had been appointed. solely because they found themselves unable to control the American Medical Association, and through it the Congress.

Rather than suffer themselves to be left out, they are willing to let the impression go abroad that the Congress is doomed to failure.

We are satisfied that these gentlemen resigned their positions under a misapprehension; it was supposed that the American Medical Association would be unable to stand their defection, and would alter its whole course rather than do without them.

But it was forgotten that times had changed since Philadelphia, New York and Boston ruled medical America; the fact was overlooked that we have a great West and a great South, and that while the whole medical world is ever ready to pay all due respect to the Eastern magnates, it is not as ready as it was to be controlled by them.

Away back in the twenties, when a few men sought to control the medical profession of England, after a while the rank and file revolted, and the warfare waxed so high that on one occasion the elder Mr. Wakeley, the champion of the masses, was *forcibly* ejected from the theatre of the College of Physicians (of which he was a member in good standing) by order of the Council.

While we have not yet come to such a pass in this country, and it is to be hoped we never shall, yet the position is somewhat similar.

A few leaders are endeavoring to autocratically govern the mass of the profession, and, apparently, stand ready to endeavor to ruin that which they cannot rule.

In the meantime, the regularly-authorized Committee of the American Mebical Association are going steadily forward-making arrangements for the Congress that *will be held in Washingthn in 1887.*

This committee is the agent of the American Medical Association; and right here we wish to say most emphatically and most clearly, *that we have no sympathy with nor support for any committee, or any set of men, as a committee or set of men*, BUT WE DO MOST POSITIVELY ASSERT THAT EVERY TRUE PHYSICIAN UNQUESTIONABLY OWES HIS ALLEGIANCE TO THE AMERICAN MEDICAL ASSOCIATION, and is in honor bound to abide by, aid, and support its decisions.

The Congress is being organized under its auspices and direction: hence every man who believes in subserviency to law and order must give his support to this organization.

If the Association at any time sees fit to reconsider its action; if it modifies or changes in toto its present attitude, such change will receive our most cordial support, as it should that of every right-minded man.

Let it be but clearly understood that the question is not that of supporting one or the other party, but of supporting or rebelling against the power of the American Medical Association; let it be realized that, unfortunately, many of our leaders are on the side of opposition; let it be appreciated that their attitude is much like that of minority leaders in South American republics, who would rather ruin than be governed by those who are distasteful to them; let these points be clearly understood, and the whole matter is plain.

There can be no two sides to this question. On the one hand are arrayed those who would support the American Medical Association; on the other, those who would crush it because it will not do their bidding.

Next spring will decide who are in the majority.

*In the meantime, let us again most positively state that the Congress will be held, and will be well worthy of attendance.*

We say amen to every word of the above editorial from the *Medical and Surgical Reporter*, of Philadelphia, one of the very foremost weekly journals of this country. The wording can be easily changed so as to apply to a lot of disgruntled dentists who are endeavoring to throttle the movements of President Taft in organizing the Oral and Dental Section.

—*Dr. B. H. Catching.*

